# Idiopathic Intracranial Hypertension in Children: Clinical Presentations and Management

**DOI:** 10.4103/0974-9233.51985

**Published:** 2008

**Authors:** Hisham A. Aboul Enein, Amr F. Abo Khair

**Affiliations:** 1 From the Department of Neurosurgery, Faculty of Medicine, Alexandria Medical School, Egypt; 2 From the Department of Ophthalmology, Faculty of Medicine, Alexandria Medical School, Egypt

**Keywords:** Idiopathic Benign intracranial hypertension, papilledema, Vision loss

## Abstract

**Background:**

Idiopathic intracranial is common in adults, particularly obese young women, but also occurs in children and adolescents.

**Aim:**

**C**linical presentation of idiopathic intracranial hypertension in the pediatric population and how the presenting signs and symptoms may be different from those seen among adult patients.

**Results:**

This study is a prospective study conducted in the Alexandria Medical School, Egypt, between the periods starting from January 2003 till December 2007. Ten patients were included in this study, 9 patients were treated with repeated spinal taps while only one patient necessitated insertion of a theco-peritoneal shunt.

**Conclusion:**

Idiopathic intracranial hypertension may occur in children as among adults. If diagnosed early, visual acuity can be saved with proper management.

Idiopathic intracranial hypertension (IIH) also known as pseudo tumor cerebri is a disorder characterized by increased intracranial pressure (ICP) in the presence of normal cerebrospinal fluid chemistry (CSF), normal neuroimaging, and no localizing signs on neurological exam (with the exception of cranial nerve VI palsy). Although this condition is known as ‘benign intracranial hypertension’ it is not truly benign in that there is the potential for permanent visual loss. The papilloedema and visual deficits associated with IIH are an ophthalmologic emergency and require prompt evaluation and treatment.^1^–^4^ The exact pathophysiological mechanism for IIH is still unknown. Suggested mechanisms have included increased CSF production, decreased CSF absorption at the level of the arachnoid granulations, or increased cerebral venous pressure.^5^–^8^ It has also been noted that there is an association between IIH and Chiari I malformation (inferior cerebellar tonsillar herniation), and it has been suggested as a secondary cause of IIH.^9^,^10^

Patients more often present with symptoms reflecting generalized increased ICP. Common initial complaints include headache and visual disturbances including diplopia or blurred vision.^11^–^14^

The aim of the present paper was to study idiopathic intracranial hypertension occurring among pediatric patients in terms of clinical presentation, sex and age distribution, associated ophthalmological abnormalities, and the prognosis following different treatment modalities.

## Material and Methods

This is a prospective study starting from January 2003 till December 2007. Ten patients suffering from pseudotumour cerebri were included in our work; the age ranged between 6 till 18 years, admitted to Alexandria main university hospital.

The clinical criteria of the patients involved in our study included:

- symptoms and signs of increased intracranial pressure
- normal neurological examinations with the exception of papilledema, visual loss, or unilateral or bilateral sixth nerve palsy cranial
- absence of a mass lesion or hydrocephalus confirmed with cranial computed tomography
- And Cerebrospinal fluid opening pressure by lumbar puncture higher than 20 cm H_2_O with normal fluid chemistry.

Thorough history taking was done with special emphasis on the age, sex, recent weight gain, medications which predispose to intracranial hypertension such as tetracycline, chronic steroids, or synthetic growth hormone, or history of any underlying medical conditions associated with pseudotumour cerebri such as Addison disease or systemic lupus erythematosus. Careful documentation of visual acuity, fundus, visual fields, and ocular motility was done to all patients as a crucial step for diagnosis and then as a prognostic tool. All patients were clinically followed up every two weeks for a period of 6 months.

**Figure 1:**
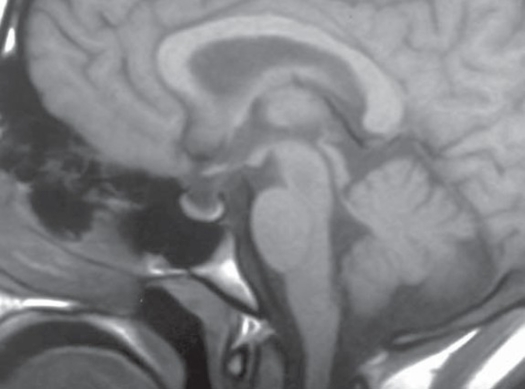
Sagital MRI T1 weighted image of the brain and sella showing features of increased intracranial pressure in a 7-year-old boy, partial empty sella syndrome.

**Figure 2:**
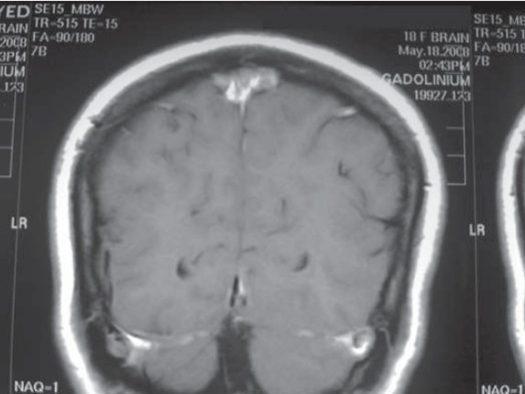
Coronal MRI showing delta sign demonstrating superior sinus thrombosis.

Computerized axial tomography of the whole brain and posterior fossa with intra venous contrast was done to all of our patients to exclude the presence of any mass lesions. MRI brain was done to all patients to exclude tonsillar herniation. MRV of the brain was done for one patient with fulminate clinical signs and symptoms where venous sinus thrombosis was suspected (Fig 1 and 2).

Lumbar spinal tap was done initially for all cases to measure the CSF opening pressure and for CSF sampling (Table 1). The patients were followed up ophthalmologically every 15 days for assessment of the visual acuity together with fundus examination. Repetition of lumbar taps was done only when medical treatment failed to control symptoms or when visual manifestations persisted. The time interval between two successive taps depended upon the response on medical therapy and fundus changes. Maximum number of spinal taps was three times. Theco-peritoneal shunt was inserted for one patient who did respond neither on medical treatment nor on repeated lumbar taps.

**Table 1:** Opening Pressure during Lumbar Taps Found amongOur Patients.

Number of Patients	Opening Pressure in cmH_2_O
1	20-30

3	30-40

4	40-50

2	More than 50

It shows that the opening pressure among our patients was commonly between 30-50 cmH_2_O.

## Results

**Figure 3:**
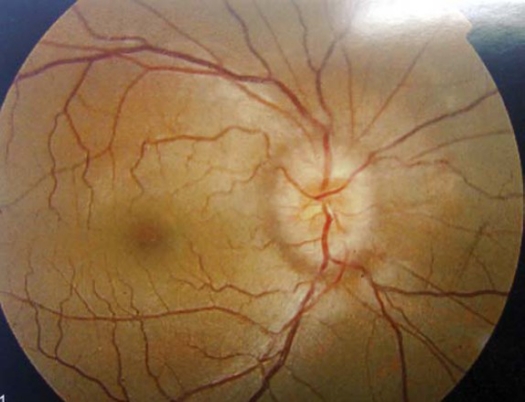
Fundus photography of 14-year-old female with IIH showing severe papilledema.

Of the 10 patients included in the present study, six were girls while only four were boys. Seven patients were in between the age of 9 to 12 years “pre-puberty age” (Table 2). The most common presenting symptoms found in this study was headache (all patients) while diplopia and unilateral abducent palsy was only found in six patients, bilateral abducent palsy was found in four patienst. Field abnormalities were detected in two patients where the blind spot was enlarged in one patient and temporal field defects in the other (Table 3). Severe visual loss resulting in chronic disc atrophy that led to post papilledemic optic atrophy was found in one eye of one patient (Fig 3). Nine patients were treated adequately medically. Only one patient who suffered severe visual problems which did not respond to neither to conservative treatment nor to repeated lumbar taps required insertion of a lumboperitoneal shunt (Table 4).

**Table 2:** Number of Patients in Each Age Group.

Number of Patients	Age in Years
2	6-9

5	9-12

2	12-15

1	15-18

Majority of the cases included in this study were in the prepubertal age.

**Table 3:** The CP among Our Patients

Presenting Symptoms & Signs	Number of Patients
Headache	10

Papilledema	10

Unilateral Abducent Palsy	6

Bilaternal Abducent Palsy	4

Field Defects	2

It shows that headache & papilledema was cardinal signs among all patients yetstrabismus was found in 4 patients.

**Table 4:** Relation between Opening Pressure and Responseto Treatment Modality.

Opening Pressure in cmH2O	Fundus Changes	Treatment Modality
20-30	Papilledema	Single lumbar tap & diuretics

30-40	Severe papilledema	Repeated lumbar tap (2times) & corticosteroids plus diuretics

40-50	Enlarged blind spot	Repeated lumbar tap (3times) & corticosteroids plus diuretics

More than 50	Pallor of the optic disc & Temporal field defects	Thecoperitoneal shunt

It shows the response on different treatment options and its relations to the CP and response to treatment modality

Follow up was done for at least 6 months up to 2 years time. Resolution of papilledema occurred rapidly in 9 patients, with a mean of 4.7 months. Resolution of sixth nerve palsy also occurred rapidly in four patients in a mean of 1.6 months. One patient had established strabismus **Graphs (1)**.

**Graph:**
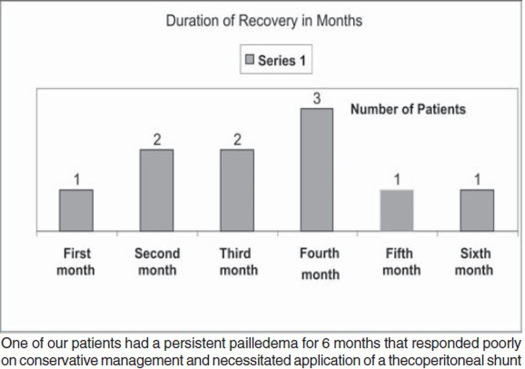
Duration of recovery from papilledema in months.

## Discussion

The results of our study conclude that female are commonly affected than males, with age prevalence for those above 10 years. Same results were also found to be true in other studies.^15^ This might be due to more obesity in adolescence. One study reports that as many as 60% of children who develop the disorder are over 10 years of age.^16^ Recent study proves that idiopathic intracranial hypertension differs from younger children than in older ones, without sex predilection.^2^,^17^ Similarly, Stiebel-Kalish et al^25^ defined males 13–15 years of age and females 11–15 years of age as pubertal in one combined age- and sex-specific criteria for idiopathic intracranial hypertension among children with a weak association between pediatric IIH and obesity.^15^,^18^

Nine of our patients responded well to repeated lumbar puncture and conservative medical therapy. Most cases of pediatric IIH respond well to adequate medical therapy thus, rendering surgical management reserved only for those who fail medication.^16^,^18^ Headaches usually resolve rapidly once reduction in cerebrospinal opening pressure happens, yet some authors report the persistence of headaches even after lumbar tap.^19^,^20^,^21^

Thecoperitoneal shunting is preferred for those children who failed to respond medically or after repeated lumbar taps and considered as the most successful operative procedure.^22^ This procedure, however, is associated with various complications including shunt obstruction, lumbar radiculopathy, infection, as well as tonsillar herniation.^23^–^25^ Children, specifically, may be at higher risk for developing complications, possibly secondary to increased mechanical stress (growth).^23^,^24^ In one report, shunts lasted only 6†months, with an average time to failure of 9†months.^4^ In another, they lasted an average of 18 months.^25^ Additionally, LP shunting has failed to†halt progressive vision loss in some cases.^24^ Unfortunately, to date there are no reliable risk factors that predict poor shunt tolerance and the long-term outcome of visual function after LP shunting.

Permanent visual loss is reported in 6–20% of pediatric cases, the severity of papilledema, particularly if pallor and cotton-wool spots are present, is positively correlated with the risk of visual loss.^2^,^15^,^20^,^26^,^27^

## Summary

Ten patients below the age of 18 years old diagnosed with manifestations of idiopathic intracranial hypertension were studied at the Alexandria medical school for both the neurological dysfunction and for ophthalmologic assessment. Nine patients recovered completely on medical therapy and repeated lumbar tap. One patient had a high intracranial pressure resistant to conservative methods and necessitated an insertion of a thecoperitoneal shunt.

## Conclusions

Idiopathic intracranial hypertension occurs among children as it occurs among adults. Female predominance is certainly proved, visual manifestations may be dramatic. Visual acuity can be saved only if proper early management is done. Repeated lumbar puncture can be of great help once diagnosis is established. Although the known complications of inserting thecoperitoneal shunt, yet it must be done for those patients with resistant forms of pseudo tumour cerebri.
